# Chinese herbal medicine treatment and the association with long-term major adverse cardiac events in patients with chronic kidney disease: A propensity-score matched cohort study

**DOI:** 10.37796/2211-8039.1699

**Published:** 2026-03-01

**Authors:** Yuan-Ching Liao, Mei-Yao Wu, Cheng-Li Lin, Chiz-Tzung Chang, Peter K. Mayer, Hung-Rong Yen

**Affiliations:** a Department of Chinese Medicine, China Medical University Hospital, Taichung 404327, Taiwan; b Graduate Institute of Chinese Medicine, School of Chinese Medicine, College of Chinese Medicine, China Medical University, Taichung 404328, Taiwan; c School of Post-baccalaureate Chinese Medicine, College of Chinese Medicine, China Medical University, Taichung 404328, Taiwan; d Management Office for Health Data, China Medical University Hospital, Taichung 404327, Taiwan; e School of Medicine, College of Medicine, China Medical University, Taichung 404328, Taiwan; f Division of Nephrology, Department of Internal Medicine, China Medical University Hospital, Taichung 404327, Taiwan; g Chinese Medicine Research Center, China Medical University, Taichung 404328, Taiwan; h Research Center for Traditional Chinese Medicine, China Medical University Hospital, Taichung 404327, Taiwan; i International Master Program of Acupuncture, College of Chinese Medicine, China Medical University, Taichung 404328, Taiwan; j School of Chinese Medicine, College of Chinese Medicine, China Medical University, Taichung 404328, Taiwan

**Keywords:** Chronic kidney disease (CKD), Chinese herbal medicine (CHM), Cardiovascular disease (CVD), Major adverse cardiac events (MACEs), National Health Insurance Research Database (NHIRD)

## Abstract

**Background:**

Cardiovascular disease (CVD) represents the leading cause of mortality among individuals with chronic kidney disease (CKD). While Chinese herbal medicine (CHM) is commonly used by CKD patients in Taiwan, the impact of CHM use on cardiovascular outcomes in this population remains insufficiently understood.

**Aims:**

This study aimed to evaluate the association between CHM use and the long-term risk of major adverse cardiac events (MACEs) in patients with CKD.

**Methods:**

Data were obtained from the Taiwan National Health Insurance Research Database to identify patients aged over 20 years with newly diagnosed CKD. A 1:1 propensity score matching was performed based on age, sex, comorbidities, medication use, and CHM exposure, resulting in 6351 matched pairs. Participants were followed from 2000 to 2017 to assess the incidence of MACEs, including heart failure (HF), myocardial infarction (MI), ischemic stroke (IS), cardiovascular death, and all-cause mortality.

**Results:**

The CHM group comprised 56.4 % females with a mean age of 49.4 ± 15.3 years. After matching, CHM use was associated with a statistically significant reduction of 23 %–31 % in the adjusted hazard ratios for various cardiovascular outcomes and all-cause mortality (P < 0.001). The most commonly prescribed CHM formula and single herb were Ji-Sheng-Shen-Qi-Wan (JSSQW) and Danshen (*Salviae Miltiorrhizae*), respectively.

**Conclusions:**

The use of CHM as an adjunct therapy in CKD patients was associated with a significantly lower risk of MACEs and all-cause mortality. These findings support the potential of CHM in cardiovascular risk mitigation among CKD patients and highlight the need for future clinical and ethnopharmacological investigations.

## 1. Introduction

Cardiovascular diseases (CVDs) have become a leading cause of death and disability globally in recent years [1]. CVDs are associated with damage to the heart and blood vessels. According to the World Health Organization, 85 % of the associated deaths were attributed to heart attacks and strokes in 2022. Individuals with chronic kidney disease (CKD) have an increased risk of developing CVD relative to the general population due to electrolyte imbalances, anemia, proteinuria, and hemodynamic factors [2].

Irreversible reductions in the estimated glomerular filtration rate (eGFR) and higher proteinuria substantially increase the risks of major adverse cardiac events (MACEs) and death, especially in patients with an eGFR below 60 mL/min/1.73 m^2^ [3]. Stage 5 CKD (eGFR: under 15 mL/min/1.73 m^2^) is associated with an up to three times higher risk of MACEs than stage 3a CKD (eGFR: 45–59 mL/min/1.73 m^2^) [4].

The prevention of CVDs through exercise, lifestyle changes, and dietary modification is crucial. Furthermore, traditional herbal medicines are often used to prevent or treat CVDs in Asian countries. In Taiwan, 45.3 % of CKD patients use Chinese herbal medicine (CHM) covered by the National Health Insurance (NHI) program [5]. Some studies suggest that non-aristolochic acid-containing CHM usage can delay the progression of CKD and decrease the risk of mortality [5–8]. However, CHMs are generally high in potassium and phosphorus ions [9]. However, evidence regarding the effect of CHM on cardiovascular risk in CKD patients remains limited.

The purpose of this study was to use the Taiwan NHI Research Database (NHIRD) to evaluate whether CHM reduces MACEs that involve hospitalization, here defined as the hospitalization rates of MI, HF, IS, and cardiovascular death in patients with CKD. In addition to the cardiovascular death, the risk of all-cause mortality was also evaluated.

## 2. Methods

### 2.1. Data source

This study was conducted using data collected from the Taiwan NHIRD, which covers over 99 % of the Taiwanese population. Data were obtained from the Longitudinal Generation Tracking Database 2000 (LGTD 2000), which is a subset of the NHIRD. LGTD 2000 randomly selected two million individuals from NHIRD and is considered to be a representative sample as it exhibits similar demographics and healthcare usage distribution as NHIRD. This study was approved by the Institutional Review Board of the China Medical University Hospital, Taiwan (IRB No: CHUH112-REC2-005). Since de-identified data were used, the requirement for informed consent was waived. This study followed the Strengthening the Reporting of Observational Studies in Epidemiology (STROBE) reporting guidelines.

### 2.2. Study cohort

We included patients with CKD aged over 20 years between January 1, 2000 and December 31, 2016 in our study. CKD was diagnosed according to the International Classification of Diseases-9th Revision (ICD-9) codes 582, 583, 584, 585, 586, and 587.

Patients were categorized into CHM or non-CHM groups according to their utilization of CHM. A procedure involving 1:1 propensity score matching of participants was conducted according to participant age, sex, monthly income, comorbidities, and medication. All patients were followed up until Dec 31, 2017, deaths, or withdrawals from the NHI program.

During the follow-up period, participants were divided into three groups according to the average number of days of CHM usage per year (less than 30 days, 31–90 days, and more than 90 days) to investigate whether CHM is associated with a duration-dependent effect.

The exclusion criteria were as follows: patients with major illness or injury records on the Taiwan Registry for Catastrophic Illness who were admitted for dialysis; patients younger than 20 years on the index date; CHM prescription before the index date; diagnosis of malignancy during the baseline observation and follow-up periods; and the presence of a previously diagnosed MI, HF, and IS or cardiovascular events within one year from the initial diagnosis date of CKD. The study participants were tracked for cardiovascular outcomes until December 31, 2017.

### 2.3. Covariates and end points

The occurrence of a MACE during the follow-up period was the primary outcome of this study. MACEs included cardiovascular mortality and cardiovascular events, which were defined as the rate of hospitalization due to HF, MI, and IS. We adopted the in hospital healthcare database to ensure inpatients had a discharge diagnosis of ICD-9-CM codes 410.X (acute MI), 428 (congestive heart failure), and 433.xx, 434.xx (IS). Cardiovascular mortality was defined according to ICD-10 codes: I01–I99. The inpatient records of the NHIRD were used to confirm that CVD was the cause of death during their hospitalization. All-cause mortality was defined as the patient being withdrawn from the NHI program.

Additionally, CKD diagnosis had to be made initially by a physician since CKD stage and laboratory data could not be obtained from the database. We used diagnostic codes and drug types used by patients to distinguish disease severity. Comorbidities including diseases of the circulatory system, endocrine and metabolic diseases, and other diseases were recorded. Medications included α-blockers, β-blockers, calcium channel blockers (CCBs), angiotensin-converting enzyme inhibitors (ACEIs) or angiotensin receptor blockers (ARBs), diuretics, digoxin, nitrates, anticoagulants, aspirin, statins, antihyperglycemic agents, insulin and erythropoiesis-stimulating agent (ESA).

### 2.4. Statistical analysis

We used the chi-square test for categorical variables, which are shown as frequencies (percentages). All continuous variables were tested using the nonparametric Wilcoxon rank-sum test, and the data are reported as the median (interquartile range). First, we adopted univariable logistic regression. The Cox proportional hazards model with adjustments for confounders was further conducted to analyze the association between MACEs and CHM. The confounding variables included age, sex, comorbidities, medications, monthly income, and urbanization level. Odds ratios (ORs) and 95 % CIs were estimated. The Kaplan–Meier method was used to estimate survival over time and cumulative incidence of hospitalization. All analyses were performed using SAS, version 9.4 (SAS Institute Inc), and R software, version 3.5.1 (R Foundation for Statistical Computing, Vienna, Austria). Two-tailed tests with P < 0.05 were considered statistically significant.

## 3. Results

**Fig. 1:**
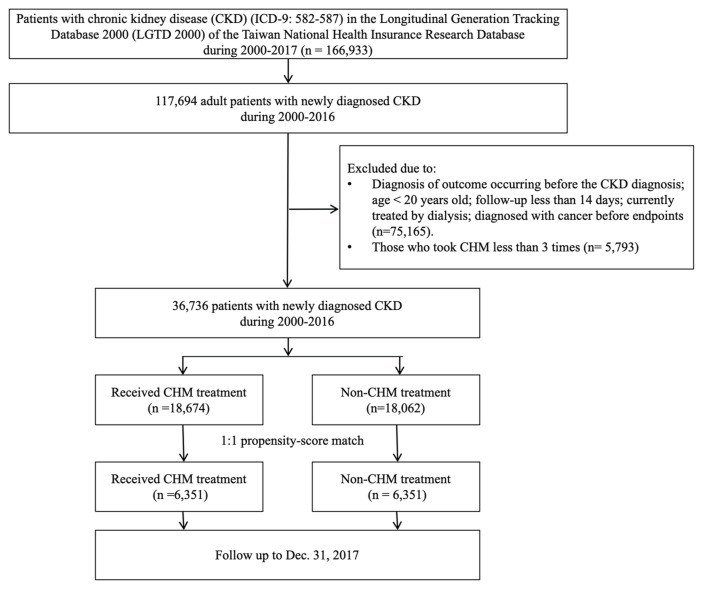
Participant inclusion flowchart using the 2000 version of the Taiwan National Health Insurance Database. Patients with newly diagnosed chronic kidney disease were included, and their cardiovascular mortality rate and incidence of hospitalization for cardiovascular events were tracked until the end of 2017. Abbreviations: CKD: chronic kidney disease, LGTD 2000: Longitudinal Generation Tracking Database 2000, CHM: Chinese herbal medicine.

From the 2 million individuals in the LGTD 2005, 117,694 were eligible based on a first-time physician diagnosis of CKD. A total of 75,165 patients were excluded due to the following: (1) being younger than 20 years of age; (2) MACEs occurring before the diagnosis of CKD; (3) a history of cancer; and (4) entering dialysis during the follow-up period. Furthermore, 5793 patients were excluded for the using CHM fewer than three times. Among the 36,736 remaining patients, 18,062 were treated only with Western medicine, and 18,674 patients used CHM and Western medicine concurrently. Fifty percent of CKD patients used CHM in this study. After 1:1 propensity score matching was conducted based on age, sex, comorbidities, and medication, a total of 6351 participants were included in each group for follow-up until Dec 31, 2017 (Fig. 1). The demographic characteristics of the patients in this study are presented in Supplementary Table 1 (https://www.biomedicinej.com/cgi/editor.cgi?window=additional_files&article=1699&context=biomedicine). We identified 36,736 patients for follow-up, including 17,099 females (46.5 %) and 19,637 males (53.5 %). The prevalence of CKD was observed to increase with age.

Among CKD patients that used CHM, female, middle-aged (40–59 years) patients living in the city and classified as middle socioeconomic status had the highest ratio of CHM users. The 1:1 matching of participants was conducted according to the participant’s age, sex, comorbidities, income, and medication history. There were no significant differences between the two groups after matching (Table 1). During the follow-up period, the CHM group had a significantly lower crude hazard ratio (HR) of hospitalization for MI (0.35; 95 % CI: 0.27, 0.46; P < 0.001) than the non-CHM group. In addition, the CHM group had a significantly lower crude HR of hospitalization for IS (HR: 0.30; 95 % CI: 0.25, 0.36; P < 0.001) and hospitalization for HF (HR: 0.35; 95 % CI: 0.29, 0.41; P < 0.001) than the non-CHM group. Crucially, the HRs of cardiovascular death (HR: 0.37; 95 % CI: 0.31, 0.45; P < 0.001) and all-cause mortality (HR: 0.28; 95 % CI: 0.26, 0.30; P < 0.001) were significantly lower in the CHM group.

After adjusting for all covariates associated with MACEs, the fully adjusted HR of hospitalization for MI in the CHM group was 0.31 (95 % CI: 0.24, 0.41; P < 0.001). The adjusted HR of hospitalization for IS was 0.25 (95 % CI: 0.2, 0.3; P = 0.01). The adjusted HR of hospitalization for HF was 0.28 (95 % CI: 0.24, 0.33, P < 0.001). The adjusted HR of cardiovascular death was 0.29 (95 % CI: 0.24, 0.36; P < 0.001). The adjusted HR of all-cause mortality was 0.23 (95 % CI: 0.21, 0.25; P < 0.001) (Table 2).

We also conducted an herbal accumulative dose analysis. With the increase in cumulative days of CHM use, the adjusted HR decreased slightly in MACE-related hospitalization and mortality rates in patients with CKD.

The Chinese herbal products most frequently prescribed by TCM doctors for treating CKD patients at risk of MACEs are presented in Supplementary Table 2 (https://www.biomedicinej.com/cgi/editor.cgi?window=additional_files&article=1699&context=biomedicine) (single herbs) and Supplementary Table 3 (https://www.biomedicinej.com/cgi/editor.cgi?window=additional_files&article=1699&context=biomedicine) (Chinese herbal formulas). Ji-Sheng-Shen-Qi-Wan (JSSQW) was the most frequently prescribed Chinese herbal product, followed by Ma-Zi-Ren-Wan (MZRW), Tian-Wang-Bu-Xin Dan (TWBXD), Zhi-Gan-Cao-Tan (ZGCT) and Zhi-Bai-Di-Huang-Wan (ZBDHW). The most commonly used single-herb TCMs included Danshen (*Salviae Miltiorrhizae*), Dahuang (*Rheum officinale, Rheum palmatum*), Huangqi (*Astragalus membranaceus*), Cheqianzi (*Plantago asiatica*), and Heshouwu (*Polygonum multiflorum* Thunb.).

Since Danshen and JSSQW were the most frequently prescribed single herb and Chinese herbal formula, we compared the HRs of MACEs between Danshen and JSSQW in patients with CKD. Danshen and JSSQW had significant effects on the risk of all-cause mortality. The adjusted HRs were 0.38 (95%CI: 0.23, 0.64; P < 0.001) and 0.39 (95 % CI: 0.25, 0.62; P < 0.001), respectively. JSSQW reduced the risk of hospitalization for HF in patients with CKD(HR: 0.34; 95 % CI: 0.14, 0.85; P < 0.001) (Table 3).

**Fig. 2:**
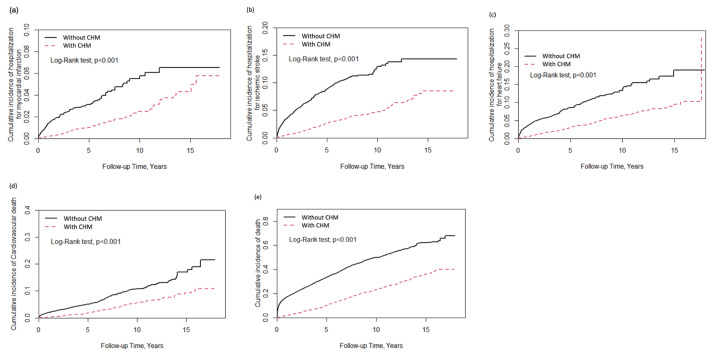
Kaplan–Meier plots showing the cumulative incidence of hospitalization and death due to the following major adverse cardiovascular events: (a) myocardial infarction, (b) ischemic stroke, (c) heart failure, (d) cardiovascular death, and (e) total death in patients with chronic kidney disease (solid line: patients only using Western medicine; dotted line: patients using Western medicine and Chinese herbal medicine concurrently.

**Fig. 3:**
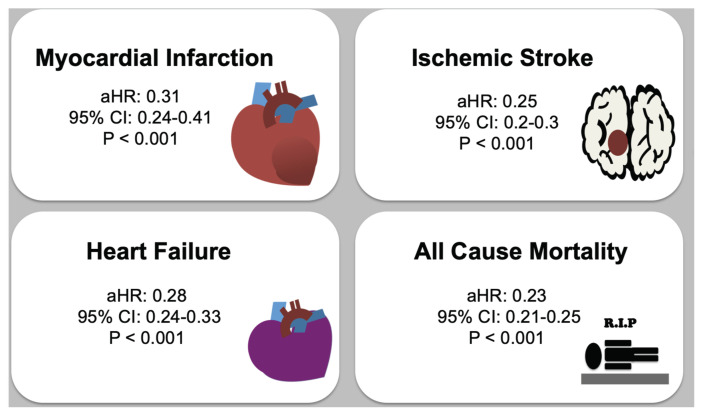
A graphical summary illustrating that Chinese herbal medicine treatment is association with a reduced risk of long-term major adverse cardiac events in patients with chronic kidney disease.

During follow-up, Kaplan–Meier analysis revealed that the CHM group had a significantly lower cumulative incidence of hospitalization for MI than the non-CHM group (log-rank test; P < 0.0001), as depicted in Fig. 2a. The results presented in Fig. 2b and c reveal that the integrated group had a significantly lower cumulative incidence of hospitalization for IS and HF than the non- CHM group (log-rank test; P < 0.0001). Fig. 2d and e reveal that CHM treatment was significantly associated with a lower risk of cardiovascular death and all-cause death in CKD patients (log-rank test; P < 0.0001). Theoretically, the incidence of non-CVD accidents should be independent of CHM treatment during the follow-up period. A sensitivity test was conducted to test the incidence of traffic accidents between two groups, and the results showed that there was no impact of CHM usage on traffic accidents in patients with CKD (data not shown). Overall, Chinese herbal medicine treatment is associated with a reduced risk of long-term major adverse cardiac events in patients with chronic kidney disease (Fig. 3).

## 4. Discussion

This is the first large-scale, population-based, retrospective cohort study to investigate the association between CHM and MACEs in patients with CKD. This study indicates that in patients with moderate to severe renal disease, the integration of CHM into the treatment approach can reduce cardiovascular events. Furthermore, cardiovascular death decreased significantly as the cumulative number of days of CHM use increased.

CKD is an important independent risk factor for CVDs, along with age, sex, and the co-occurrence of hypertension, diabetes, and/or hyperlipidemia [10]. Therefore, factors such as age, sex, comorbidities, and medication usage were adjusted to eliminate possible interference factors in this study. Additionally, we adjusted for the salary range and urbanization level to decrease the influence of socioeconomic status on MACEs [11].

CKD patients are at a high risk of experiencing cardiovascular events, and the increase in the severity of CKD leads to an increase in cardiovascular events and hospitalization risk [3]. However, CKD stage and laboratory data could not be obtained from the NHI database, we tried to use other information as surrogate disease severity markers. Patients who were admitted for dialysis were excluded using injury records from the Taiwan Registry for Catastrophic Illness. We used diagnostic codes to ensure that included participants had an estimated glomerular filtration rate (eGFR) below 60 mL/min/1.73 m^2^ and had not received dialysis treatment.

One of the key pieces of information is the type of prescribed medication, which could be used to distinguish the severity of CKD. For example, metformin has been contraindicated in patients with impaired kidney function due to the risk of lactic acidosis [12], and the ESA is prescribed for patients with a serum creatinine level of >6.0 mg/dL and hematocrit of <28 % under the NHI program in Taiwan. These parameters are approximately equivalent to stage 5 CKD [13,14].

For individuals with CKD, the management of blood pressure, blood sugar, and cholesterol is important for reducing CVD risk. There are favorable or unfavorable cardiovascular effects associated with different medications. Renin-angiotensin system (RAS) inhibitor therapy and CCBs may offer similar cardiovascular protection in patients with advanced CKD [15]. There is a strong association between insulin treatment with the risk of MACEs in type 2 diabetes mellitus (T2DM) patients [16]. In contrast, sodium/glucose cotransporter 2 (SGLT2) inhibitors may ameliorate CVD risk in T2DM patients with an eGFR of 30 to <90 mL/min/1.73 m^2^ [17]. Therefore, we adjusted for the variables in the drug category to minimize heterogeneity in the two study groups.

To better assess the potential long-term effects of CHM on MACE-related hospitalizations, we used average annual CHM exposure as the primary criterion, rather than simply relying on cumulative days of use. Including individuals with fewer than 30 days of CHM use per year may attenuate the observed treatment effect. However, our analysis stratified patients by cumulative annual exposure, which allowed us to explore a potential dose-response relationship. Among the 6351 patients in the CHM group, 38.9 % used CHM for fewer than 30 days per year, 27.8 % used it for 30–60 days annually, and 33.2 % used it for more than 90 days per year. This distribution supports the possibility of a dose-dependent association between CHM use and cardiovascular outcomes.

From this study, we observed that the use of CHM significantly reduced the incidence of all cardiovascular events, including the hospitalization rates for MI, HF, and IS in CKD patients. Additionally, CHM use significantly reduced not only the total mortality rate but also cardiovascular death. Moreover, the escalation in cumulative days of CHM usage demonstrated a noteworthy impact in lowering the occurrence of MACEs.

Previous studies have reported that middle-aged patients most frequently use CHM in Taiwan [18]. In our study, 45.5 % of CKD patients taking CHM were between 40 and 59 years of age. Due to several lifestyle factors such as a different social culture, work schedule, work stress, and excessively long working hours, the incidence of cardiovascular events is higher among Taiwanese workers aged 45–55 [19]. In other words, an age effect that associates middle-aged workers in Asia with an increased cardiovascular risk due to work stress is observed [20]. Therefore, the present study observed middle-aged patients with declining renal function and continued to track them for many years.

In the population-based study, CHMs targeting CKD and MACEs included Danshen (*Salviae Miltiorrhizae*), Dahuang (*Rheum officinale, Rheum palmatum*), Huangqi (*Astragalus membranaceus*), Cheqianzi (*Plantago asiatica*), and Heshouwu (*Polygonum multiflorum* Thunb.). The most commonly used herbal medicines are JSSQW, MZRW, TWBXD, ZGCT, and ZBDHW.

Danshen is widely used for cardiovascular and cerebrovascular diseases and to alleviate kidney and liver-related diseases [21]. The combination of Danshen and aspirin or clopidogrel and can influence metabolic pathways, including those involved in lipid metabolism, phospholipid metabolism, and hemorheology [22,23]. Danshen, when used as an add-on antihypertensive therapy, reduces systolic blood pressure in Taiwanese patients with uncontrolled hypertension [24].

Huangqi has antioxidative, antihyperglycemic, and immune-regulating effects and is often used in remediating diabetic nephropathy with proteinuria. It has a two-way regulating effect on blood pressure [25], which can prevent myocardial ischemia and myocardial hypertrophy and delay the course of HF [26]. Huangqi combined with antihypertensive drugs is more effective in improving systolic blood pressure, microalbuminuria, and serum creatinine [27].

Heshouwu has antihyperlipidemic, antiatherosclerotic, antioxidant, and anti-inflammatory effects [28]. The active ingredient of Heshouwu, tetrahydroxystilbene glucoside, can alleviate cerebral ischemic injuries and pressure overload-induced cardiac remodeling [29,30].

Due to reduced erythropoietin production and iron deficiency, patients with moderate to severe CKD always suffer from anemia [31]. Anemia is associated with increased cardiovascular mortality in CKD patients [32]. Danshen and Huangqi are widely known CHMs in Asia. Both herbs are used for the associated protective effects against anemia-related mortalities [33].

JSSQW and Danshen are used extensively in CKD, and they can improve the long-term survival rate of patients [8]. JSSQW has a diuretic and inotropic effect. TCM physicians often prescribe JSSQW for patients with HF and predialysis nephropathy [34]. Fuzi (*Aconitum carmichaelii*) in JSSQW can improve left ventricular ejection fraction (LVEF) and lower plasma N-terminal pro B type natriuretic peptide (NT-proBNP). In addition, it can improve overall quality of life and reduce the rate of HF–related readmission [35].

ZGCT combined with antiarrhythmic drugs had a significant effect on improving premature ventricular contractions and bradycardia [36,37]. Moreover, ZGCT combined with Western medicine was shown to have a stronger therapeutic effect on LVEF and NT-proBNP in patients with chronic HF than Western medicine alone [38]. Previous studies indicate that ZGCT and Danshen are not only the most frequently used prescriptions for patients with HF in Taiwan but are also used by patients after being discharged from the hospital for acute MI [39,40].

Although the use of CHM to prevent MACEs in patients with CKD remains controversial, this population-based study in Taiwan suggests that combining Western medicine with CHM may be associated with cardiovascular benefits to cardiovascular health in patients with CKD. Specifically, this combination can prevent hospitalization due to cardiovascular events and reduce cardiovascular mortality. The participants we included had a high prevalence of comorbid hypertension (52.6 %) and hyperlipidemia (43.3 %). The combined use of CHM may potentially improve the severity of blood pressure and lipid levels, leading to better outcomes. However, because of the multiple active components of CHM and the possible synergistic pharmacological effects of combining CHM and Western medicine, rigorous clinical trials are required to confirm the cardioprotective effects of CHM in patients with moderate to severe CKD.

This study had some limitations. First, CKD stage, laboratory data, biopsy or ultrasound could not be obtained from the Taiwan health insurance database. We are unable to identify the stage of chronic kidney disease in patients or determine the exact cause of chronic kidney disease, such as glomerulonephritis, autoimmune disease, UTIs, PKD, etc. Due to the unknown actual cause of the disease and the unavailability of clinical parameters such as the severity of proteinuria, patient GFR, blood pressure, blood glucose, and blood lipid levels, disease severity among patients was distinguished based on diagnostic codes, major injury codes, and drug types. Therefore, the severity of chronic kidney disease between the two groups of patients is comparable. We carefully drew conclusions while acknowledging the study’s limitations. Moreover, these items were reviewed by the NHI Administration, thus increasing the rigor of this research. Second, because we did not have information on the patient lifestyle, we assigned lifestyle classifications based on salary range and urbanization level. Third, some studies suggest that CHM users exhibit increased health-seeking behaviors. In the current study, we could not determine whether the CHM group had a lower occurrence of risk factors for cardiovascular events, such as alcoholism, smoking, obesity, or higher levels of daily exercise. Fourth, we discussed that common comorbidities in chronic kidney disease patients, combined with the use of CHM, may affect the incidence of MACEs. However, mental health disorders, infections, or poor compliance may affect cardiovascular health. Selection bias may be present in the model. A more rigorous prospective cohort study needs to be conducted to confirm the effect of CHM intervention.

## 5. Conclusions

Combining conventional Western medicine with CHM for patients with CKD may reduce long-term MACEs, including the hospitalization rate for cardiovascular events such as CHF, MI, IS, cardiovascular death, and all-cause mortality. Further clinical trials based on these findings are warranted.

## Supplementary Information



## Figures and Tables

**Table 1:** Demographic characteristics of patients, including sex, age, financial status, urbanization level, comorbidities, and drug use after 1:1 matching.

Variable	After 1:1 matching				

	Non-CHM (N = 6351)		CHM (N = 6351)		p value
		
n	%	n	%	
Sex					0.97
Female	2651	41.7	2649	41.7	
Male	3700	58.3	3702	58.3	
Age group					0.99
20–29	345	5.43	354	5.57	
30–39	695	10.9	709	11.2	
40–49	1120	17.6	1133	17.8	
50–59	1534	24.2	1521	24	
60–69	1362	21.5	1333	21	
70–79	971	15.3	987	15.5	
>80	324	5.1	314	4.94	
Mean ± SD	55.6	15.4	55.5	15.4	0.51
Monthly income					0.98
<20,000	1729	27.2	1725	27.2	
20,000–40000	3307	52.1	3303	52.1	
≥40,000	1315	20.7	1323	20.8	
Urbanization level					0.34
1	3264	51.4	3364	53	
2	2465	38.8	2394	37.7	
3	509	8.01	481	7.57	
4	113	1.78	112	1.76	
Comorbidity					
Diseases of circulatory system					
Cardiomyopathy	4	0.06	9	0.14	0.17
Cardiac arrhythmias	497	7.83	493	7.76	0.89
Valvular heart disease	209	3.29	214	3.37	0.8
Pulmonary embolism	18	0.28	18	0.28	0.99
Deep vein thrombosis	49	0.77	50	0.79	0.92
Peripheral vascular disease	174	2.74	171	2.69	0.87
Hypertension	3341	52.6	3319	52.3	0.7
Endocrine and metabolic diseases					
Gout	1559	24.6	1563	24.6	0.93
Diabetes mellitus	2159	34	2136	33.6	0.67
Hyperlipidemia	2751	43.3	2714	42.7	0.51
Disorders of thyroid gland	372	5.86	386	6.08	0.6
Other diseases					
Liver disease	2400	37.8	2362	37.2	0.49
Chronic obstructive pulmonary disease	966	15.2	966	15.2	0.99
Peptic ulcer disease	2601	41	2563	40.4	0.49
Medications					
Alpha blocker	2287	36	2270	35.7	0.75
Beta blocker	3435	54.1	3421	53.9	0.8
CCB	3355	52.8	3345	52.7	0.86
ACEI	2171	34.2	2170	34.2	0.99
ARB	2310	36.4	2274	35.8	0.51
Diuretics	3553	55.9	3508	55.2	0.42
Antiplatelets	2207	34.8	2161	34	0.39
Anticoagulants	441	6.94	425	6.69	0.57
Digoxin	177	2.79	163	2.57	0.44
Nitrate	784	12.3	775	12.2	0.81
Aspirin	1840	29	1796	28.3	0.39
Statins	2015	31.7	1976	31.1	0.46
Fibrates	1096	17.3	1066	16.8	0.48
AGI	642	10.1	612	9.64	0.37
DPP4	542	8.53	516	8.12	0.4
TZD	585	9.21	569	8.96	0.62
Meglitinides	479	7.54	469	7.38	0.74
Sulfonylurea	1601	25.2	1590	25	0.82
Biguanides	1674	26.4	1657	26.1	0.73
SGLT2i	8	0.13	11	0.17	0.49
GLP1RA	5	0.08	7	0.11	0.56
Hypoglycemic formula	105	1.65	99	1.56	0.67
Insulin	2717	42.8	2668	42	0.38
ESA	291	4.58	286	4.5	0.83

Abbreviations: CCB: calcium channel blocker, ACEI: angiotensin-converting enzyme inhibitor, ARB: angiotensin II receptor blocker, AGI: alpha-glucosidase inhibitor, DPP4: dipeptidyl peptidase 4 inhibitor, TZD: thiazolidinedione, SGLT2i: sodium-glucose co-transporter 2 inhibitor; GLP1RA: glucagon-like peptide-1 receptor agonist, ESA: erythropoiesis-stimulating agent.

**Table 2:** Comparison of the hazard ratios for MACE-related hospitalization and mortality rates between the non-CHM group and CHM group in patients with CKD.

Variable	Cardiovascular event			Crude	p value	Adjusted^†^	p value
		
	N	Event	IR	HR (95 % CI)		HR (95 % CI)	
**Hospitalization for myocardial infarction**							
Non-CHM users	6351	141	7.41	1.00 (reference)		1.00 (reference)	
CHM users	6351	83	2.47	0.35 (0.27, 0.46)	<0.001	0.31 (0.24, 0.41)	<0.001
Cumulative number of days per year							
<30 days	2472	35	3.25	0.46 (0.32, 0.66)	<0.001	0.36 (0.25, 0.53)	<0.001
30–90 days	1768	27	3	0.42 (0.28, 0.64)	<0.001	0.40 (0.26, 0.60)	<0.001
>90 days	2111	21	1.51	0.22 (0.14, 0.35)	<0.001	0.21 (0.13, 0.33)	<0.001
**Hospitalization for ischemic stroke**							
Non-CHM users	6351	368	19.4	1.00 (reference)		1.00 (reference)	
CHM users	6351	175	5.2	0.30 (0.25, 0.36)	<0.001	0.25 (0.20, 0.30)	<0.001
Cumulative number of days per year							
<30 days	2472	74	6.87	0.38 (0.29, 0.48)	<0.001	0.29 (0.22, 0.37)	<0.001
30–90 days	1768	46	5.12	0.29 (0.21, 0.40)	<0.001	0.23 (0.17, 0.32)	<0.001
>90 days	2111	55	3.96	0.24 (0.18, 0.32)	<0.001	0.21 (0.16, 0.28)	<0.001
**Hospitalization for heart failure**							
Non-CHM users	6351	380	20	1.00 (reference)		1.00 (reference)	
CHM users	6351	212	6.3	0.35 (0.29, 0.41)	<0.001	0.28 (0.24, 0.33)	<0.001
Cumulative number of days per year							
<30 days	2472	90	8.36	0.44 (0.35, 0.56)	<0.001	0.32 (0.26, 0.41)	<0.001
30–90 days	1768	62	6.9	0.37 (0.29, 0.49)	<0.001	0.30 (0.23, 0.40)	<0.001
>90 days	2111	60	4.32	0.24 (0.18, 0.32)	<0.001	0.22 (0.17, 0.29)	<0.001
**Cardiovascular death**							
Non-CHM users	6351	291	12.4	1.00 (reference)		1.00 (reference)	
CHM users	6351	175	4.97	0.37 (0.31, 0.45)	<0.001	0.29 (0.24, 0.36)	<0.001
Cumulative number of days per year							
<30 days	2472	68	5.95	0.44 (0.34, 0.57)	<0.001	0.32 (0.25, 0.42)	<0.001
30–90 days	1768	52	5.52	0.41 (0.30, 0.55)	<0.001	0.32 (0.24, 0.43)	<0.001
>90 days	2111	55	3.84	0.29 (0.22, 0.39)	<0.001	0.25 (0.18, 0.33)	<0.001
**All-cause mortality**							
Non-CHM users	6351	2074	88.5	1.00 (reference)		1.00 (reference)	
CHM users	6351	876	24.9	0.28 (0.26, 0.30)	<0.001	0.23 (0.21, 0.25)	<0.001
Cumulative number of days per year							
<30 days	2472	361	31.6	0.34 (0.30, 0.38)	<0.001	0.26 (0.23, 0.29)	<0.001
30–90 days	1768	254	27	0.30 (0.26, 0.34)	<0.001	0.24 (0.21, 0.28)	<0.001
>90 days	2111	261	18.2	0.21 (0.19, 0.24)	<0.001	0.18 (0.16, 0.21)	<0.001

Abbreviations: CHM, Chinese herbal medicine; Crude HR, crude hazard ratio; 95 % CI, 95 % confidence interval.

**Table 3:** Comparison of hazard ratios of MACE-related hospitalization and mortality rates between Danshen and Ji-Sheng-Shen-Qi-Wan in patients with chronic kidney disease.

Variable	Crude HR (95 % CI)	p value	Adjusted HR (95 % CI)	p value
**Hospitalization for myocardial infarction**				
Non-CHM users	1.00 (reference)		1.00 (reference)	
CHM users				
Danshen	1.24 (0.31, 5.03)	0.76	0.61 (0.14, 2.61)	0.51
Ji-Sheng-Shen-Qi-Wan	0.46 (0.06, 3.29)	0.44	0.20 (0.03, 1.48)	0.11
**Hospitalization for ischemic stroke**				
Non-CHM users	1.00 (reference)		1.00 (reference)	
CHM users				
Danshen	0.24 (0.03, 1.73)	0.16	0.21 (0.03, 1.52)	0.12
Ji-Sheng-Shen-Qi-Wan	0.37 (0.09, 1.47)	0.16	0.26 (0.06, 1.07)	0.06
**Hospitalization for heart failure**				
Non-CHM users	1.00 (reference)		1.00 (reference)	
CHM users				
Danshen	0.46 (0.12, 1.85)	0.28	0.87 (0.36, 2.10)	0.75
Ji-Sheng-Shen-Qi-Wan	0.21 (0.05, 0.84)	0.03	0.34 (0.14, 0.85)	0.02
**Cardiovascular death**				
Non-CHM users	1.00 (reference)		1.00 (reference)	
CHM users				
Danshen	0.60 (0.15, 2.42)	0.48	0.41 (0.10, 1.68)	0.21
Ji-Sheng-Shen-Qi-Wan	0.67 (0.22, 2.08)	0.49	0.39 (0.12, 1.25)	0.11
**All-cause mortality**				
Non-CHM users	1.00 (reference)		1.00 (reference)	
CHM users				
Danshen	0.66 (0.39, 1.11)	0.12	0.38 (0.22, 0.64)	<0.001
Ji-Sheng-Shen-Qi-Wan	0.65 (0.41, 1.02)	0.06	0.39 (0.25, 0.62)	<0.001

Abbreviations: CHM, Chinese herbal medicine; Crude HR, crude hazard ratio; 95 % CI, 95 % confidence interval.

## Data Availability

The data that support the findings of this study are available within the article and its supplementary material.

## Abbreviations

ACEIs: angiotensin-converting enzyme inhibitors
ARBs: angiotensin receptor blockers
CCBs: calcium channel blockers
CHM: Chinese herbal medicine
CI: confidence interval
CKD: chronic kidney disease
CVDs: cardiovascular diseases
eGFR: estimated glomerular filtration rate
ESA: erythropoiesis-stimulating agent
HF: heart failure
ICD-9: *International Classification of Diseases-*9th *Revision;* IS, ischemic stroke
JSSQW: Ji-Sheng-Shen-Qi-Wan
LGTD 2000: Longitudinal Generation Tracking Database 2000
LVEF: left ventricular ejection fraction
MACEs: major adverse cardiac events
MI: myocardial infarction
MZRW: Ma-Zi-Ren-Wan
HR: crude hazard ratio
NHI: National Health Insurance
NHIRD: National Health Insurance Research Database
NT-proBNP: N-terminal pro B type natriuretic peptide
SGLT2: sodium/glucose cotransporter 2
T2DM: type 2 diabetes mellitus
TWBXD: Tian-Wang-Bu-Xin Dan
ZBDHW: Zhi-Bai-Di-Huang-Wan
ZGCT: Zhi-Gan-Cao-Tang
