# Supplementary material for: Chinese herbal medicine treatment and the association with long-term major adverse cardiac events in patients with chronic kidney disease: A propensity-score matched cohort study
Source: Biomedicine (Taipei). 2026 Mar 1;16(1):12–23. doi: 10.37796/2211-8039.1699 (PMC12962764; doi:10.37796/2211-8039.1699)
Supplement: Supplementary file 1 [file bmed-16-01-012-s001.docx]

**Supplementary Table 1.** Demographic information patients, including sex, age, financial status, urbanization level, comorbidities, and drug use.

|  | Non-CHM (N =18,062) | | CHM  (N =18,674) | |  |
| --- | --- | --- | --- | --- | --- |
| Variable | n | % | n | % | p value |
| Sex |  |  |  |  | <0.001 |
| Female | 6,574 | 36.4 | 10,525 | 56.4 |  |
| Male | 11,488 | 63.6 | 8,149 | 43.6 |  |
| Age group |  |  |  |  | <0.001 |
| 20-29 | 798 | 4.42 | 2,110 | 11.3 |  |
| 30-39 | 1,615 | 8.94 | 3,148 | 16.9 |  |
| 40-49 | 2,653 | 14.7 | 4,235 | 22.7 |  |
| 50-59 | 3,882 | 21.5 | 4,240 | 22.7 |  |
| 60-69 | 3,603 | 20 | 2,893 | 15.5 |  |
| 70-79 | 3,086 | 17.1 | 1,601 | 8.57 |  |
| >80 | 2,425 | 13.4 | 447 | 2.39 |  |
| Mean ± SD | 59.6 | ±16.9 | 49.4 | ±15.3 | <0.001 |
| Monthly income |  |  |  |  | <0.001 |
| <20000 | 5,883 | 32.6 | 3,996 | 21.4 |  |
| 20000-40000 | 8,873 | 49.1 | 9,940 | 53.2 |  |
| >=40000 | 3,306 | 18.3 | 4,738 | 25.4 |  |
| Urbanization level |  |  |  |  | <0.001 |
| 1 | 9,071 | 50.2 | 10,322 | 55.3 |  |
| 2 | 7,119 | 39.4 | 6,827 | 36.6 |  |
| 3 | 1,528 | 8.46 | 1,251 | 6.7 |  |
| 4 | 344 | 1.9 | 274 | 1.47 |  |
| Comorbidity |  |  |  |  |  |
| Diseases of circulatory system | |  |  |  |  |
| Cardiomyopathy | 53 | 0.29 | 15 | 0.08 | <0.001 |
| Cardiac arrhythmias | 1,561 | 8.64 | 1,229 | 6.58 | <0.001 |
| Valvular heart disease | 656 | 3.63 | 604 | 3.23 | 0.04 |
| Pulmonary embolism | 62 | 0.34 | 23 | 0.12 | <0.001 |
| Deep vein thrombosis | 213 | 1.18 | 104 | 0.56 | <0.001 |
| Peripheral vascular disease | 441 | 2.44 | 246 | 1.32 | <0.001 |
| Hypertension | 11,082 | 61.4 | 7,085 | 37.9 | <0.001 |
| Endocrine and metabolic diseases |  |  |  |  |  |
| Gout | 4,996 | 27.7 | 3,321 | 17.8 | <0.001 |
| Diabetes mellitus | 7,296 | 40.4 | 4,276 | 22.9 | <0.001 |
| Hyperlipidemia | 8,100 | 44.9 | 6,856 | 36.7 | <0.001 |
| Disorders of thyroid gland | 954 | 5.28 | 1,437 | 7.7 | <0.001 |
| Other diseases |  |  |  |  |  |
| Liver disease | 6,601 | 36.6 | 6,487 | 34.7 | <0.001 |
| Chronic obstructive pulmonary disease | 3,159 | 17.5 | 2,417 | 12.9 | <0.001 |
| Peptic ulcer disease | 7,401 | 41 | 7,194 | 38.5 | <0.001 |
| Medications |  |  |  |  |  |
| Alpha blocker | 7,546 | 41.8 | 4,875 | 26.1 | <0.001 |
| Beta blocker | 10,562 | 58.5 | 8,264 | 44.3 | <0.001 |
| CCB | 11,343 | 62.8 | 6,781 | 36.3 | <0.001 |
| ACEI | 7,251 | 40.2 | 4,535 | 24.3 | <0.001 |
| ARB | 8,226 | 45.5 | 4,213 | 22.6 | <0.001 |
| Diuretics | 11,498 | 63.7 | 8,003 | 42.9 | <0.001 |
| Antiplatelets | 6,991 | 38.7 | 5,395 | 28.9 | <0.001 |
| Anticoagulants | 2,154 | 11.9 | 806 | 4.32 | <0.001 |
| Digoxin | 736 | 4.07 | 287 | 1.54 | <0.001 |
| Nitrate | 3,057 | 16.9 | 1,464 | 7.84 | <0.001 |
| Aspirin | 5,987 | 33.2 | 4,160 | 22.3 | <0.001 |
| Statins | 7,863 | 43.5 | 4,996 | 26.8 | <0.001 |
| Fibrates | 3,498 | 19.4 | 2,213 | 11.9 | <0.001 |
| AGI | 2,308 | 12.8 | 976 | 5.23 | <0.001 |
| DPP4 | 2,256 | 12.5 | 703 | 3.76 | <0.001 |
| TZD | 2,108 | 11.7 | 930 | 4.98 | <0.001 |
| Meglitinides | 2,082 | 11.5 | 727 | 3.89 | <0.001 |
| Sulfonylurea | 5,689 | 31.5 | 2,844 | 15.2 | <0.001 |
| Biguanides | 5,855 | 32.4 | 2,906 | 15.6 | <0.001 |
| GLP1RA | 32 | 0.18 | 10 | 0.05 | <0.001 |
| Hypoglycemic formula | 460 | 2.55 | 139 | 0.74 | <0.001 |
| Insulin | 8,479 | 46.9 | 6,717 | 36 | <0.001 |
| ESA | 1,467 | 8.12 | 639 | 3.42 | <0.001 |

Abbreviations: CCB: calcium channel blocker, ACEI: angiotensin-converting enzyme inhibitor, ARB: angiotensin II receptor blocker, AGI: alpha-glucosidase inhibitor, DPP4: dipeptidyl peptidase 4 inhibitor, TZD: thiazolidinedione, GLP1RA: glucagon-like peptide-1 receptor agonist, ESA: erythropoiesis-stimulating agent

**Supplementary Table 2**. The five most common single traditional Chinese medicines used by patients with chronic kidney disease and relevant to the incidence and mortality of cardiovascular events.

| **Chinese herbs** | **Latin name** | **Frequency** | **Number of person-days** | **Average daily dose** | **Average duration for prescription** |
| --- | --- | --- | --- | --- | --- |
|  |  |  |  | **(g)** | **(days)** |
| Danshen | *Salviae Miltiorrhizae* | 465 | 4509 | 1.2 | 9.7 |
| Dahuang | *Rheum officinale*, *Rheum palmatum* | 286 | 2864 | 0.7 | 10 |
| Huangqi | *Astragalus membranaceus* | 263 | 2627 | 1.6 | 10 |
| Cheqianzi | *Plantago asiatica* | 202 | 2555 | 2.1 | 12.6 |
| Heshouwu | *Polygonum multiflorum Thunb.* | 154 | 2262 | 1.1 | 14.7 |

**Supplementary Table 3**. The five most common compound traditional Chinese medicines used by patients with chronic kidney disease and relevant to the incidence and mortality of cardiovascular events.

| **Chinese herbal formula** | | | | **Frequency** | | **Number of person-days** | | **Average**  **daily dose** | | **Average duration for prescription** |
| --- | --- | --- | --- | --- | --- | --- | --- | --- | --- | --- |
| **English name** | **Ingredient herbs**  **(Chinese Materia Medica name)** | |  | |  | | **(g)** | | **(days)** | |
| Ji-Sheng-Shen-Qi-Wan  (JSSQW) | | Radix Rehmanniae Praeparatae, Fructus Cornus, Rhizoma Dioscorea, Poria, Cortex Moutan, Rhizoma Alismatis, Radix Aconiti Lateralis preparata, Cortex Cinnamomi, Radix Achyranthis bidentatae, Semen Plantagines | | 415 | | 4,783 | | 4.9 | | 11.5 |
| Ma-Zi-Ren-Wan  (MZRW) | | Fructus Cannabis, Radix Paeoniae alba, Fructus Aurantii immaturus, Rhizoma Rhei, Cortex Magnoliae officinalis, Semen Armeniacae amarum | | 189 | | 2,409 | | 3 | | 12.7 |
| Tian-Wang-Bu-Xin-Dan  (TWBXD) | | Radix Rehmanniae Praeparatae, Poria, Radix Polygalae, Rhizoma Acori Graminei, Radix Scrophulariae, Semen Platycladi, Radix Platycodi, Radix Asparagi, Radix Salviae miltiorrhizae, Semen Ziziphi, Radix Glycyrrhizae, Radix Ophiopogonis, Radix Stemonae, Cortex Eucommiae, Sclerotium Poriae Circum Radicem Pini, Fructus Schisandrae, Radix Angelicae Sinensis, Radix Ginseng | | 176 | | 2,250 | | 3.5 | | 12.8 |
| Zhi-Gan-Cao-Tan  (ZGCT) | | Radix Rehmanniae Praeparatae, Fructus Cannabis, Rhizoma Zingiberis recens, Radix Ophiopogonis, Colla Corii Asini, Radix Glycyrrhizae preparata, Fructus Jujubae, Ramulus Cinnamomi, Radix Ginseng | | 162 | | 2,020 | | 3.8 | | 12.5 |
| Zhi-Bai-Di-Huang-Wan  (ZBDHW) | | Radix Rehmanniae Praeparatae, Fructus Cornus, Rhizoma Dioscorea, Poria, Cortex Moutan, Rhizoma Alismatis, Rhizoma Anemarrhenae, Cortex Phellodendri | | 155 | | 1,829 | | 4.6 | | 11.8 |
